# Multidimensional school features associated with physical activity among youth at risk of obesity: an exploratory principal component and generalized estimating equation analysis

**DOI:** 10.1186/s12889-023-16889-w

**Published:** 2023-10-16

**Authors:** Madeleine Bird, Tracie A. Barnett, Daniel Fuller, Deanna Chinerman, Marie-Ève Mathieu, Geetanjali D. Datta

**Affiliations:** 1 Public Health Agency of Canada, Office of International Affairs for the Health Portfolio, Ottawa, ON Canada; 2 Department of Family Medicine, McGill University, 5858 Côte-Des-Neiges Rd, Montreal, QC H3S 1Z1 Canada; 3 Department of Community Health & Epidemiology, College of Medicine, University of Saskatchewan, Saskatoon, Canada; 4 Department of Kinesiology, CHU Sainte-Justine, University of Montreal, 3175 Chemin de La Côte-Sainte-Catherine, Montréal, QC H3T 1C5 Canada; 5 School of Kinesiology and Physical Activity Sciences, Faculty of Medicine, Université de Montréal, Montreal, Canada; 6 Department of Social and Preventive Medicine, School of Public Health, University of Montreal, 7101 Avenue du Parc, 3E Étage, Montréal, QC H3N 1X9 Canada; 7 Department of Medicine, Cedars-Sinai Medical Center, PDC Green, 700 N San Vicente Blvd5Th Fl, West Hollywood, CA 90069 USA

**Keywords:** Schools, Built environment, Social environment, Paediatric, Physical activity, QUALITY cohort

## Abstract

**Background:**

Schools may be high-leverage points for the promotion of physical activity (PA), yet little is known about school built and social environments among youth at high risk of obesity.

**Purpose:**

To characterise school built and social environments that may be salient for PA and to examine associations between school PA environments and PA in youth at risk of obesity.

**Methods:**

Data from youth attending 206 schools (314 youth in 2005–2008, and 129 youth in 2008–2010) within the QUALITY cohort study, a longitudinal investigation of youth at high risk of obesity were used. Features of schools, based on built, policy/programming and social environments were identified using principal components (PC) analysis. Gender-stratified generalized estimating equation (GEE) models were used to explore associations between school features and accelerometer measured mean counts per minute (MCPM), mean daily moderate-to-vigorous physical activity (MVPA) and the odds of meeting MVPA guidelines cross-sectionally and prospectively using 90% confidence intervals.

**Results:**

Nine PCs were identified. Associations were observed between PA and 7 of the 9 PCs. The social environment seemed to be particularly important. *Social Norms to Promote PA* was associated with an increase in girls’ baseline MCPM and MVPA. *High Willingness to Promote PA* was associated with boys’ MCPM, MVPA, and odds of meeting MVPA guidelines, at both baseline and follow-up.

**Conclusion:**

School built and social contexts may be associated with PA cross-sectionally and over time. Further studies are necessary to confirm the direction and magnitude of effects and to establish their relevance to school-based health promotion efforts.

Physical activity (PA) is essential for the promotion and maintenance of health at all ages [1]. PA among school-aged children [2] is associated with lower blood pressure, favorable bone mineral density and cardiometabolic profiles [3], as well as favorable academic outcomes [4]. Despite the multitude of health benefits PA provides, only 9% of Canadian children between 5 and 17 years of age meet the guidelines of 60 min daily of moderate-to-vigorous physical activity (MVPA) [5, 6]. Although meeting the guidelines provides the most health benefits, even modest amounts of PA are associated with improved health outcomes [3]. Studies have found health benefits associated with meeting half of the recommended daily physical activity for youth, an average of thirty-minutes of aerobic exercise per day, including increased insulin resistance [3]. Thus, even slight improvements to PA levels among children has the potential to accrue large health benefits at the population level.

The Social Ecology Model for Health Promotion posits that within an individual’s life situation, certain environmental conditions can exert a disproportionate influence on her or his well-being [7]. The school setting is considered a credible source of information for children [8], children spend a significant proportion of their weekday time at school, and schools may also be relevant because adults and fellow peers may encourage and model positive PA behaviours that may not be found within the family context [9]. Yet, the kind of influence that a school setting may provide for PA, such as the infrastructure, environmental setting, policies and role-modeling found therein, varies considerably between schools [10]. Given that schools can be considered a high-impact leverage point, areas within a larger system where small changes can have significant impacts [7], for children’s well-being, they are an important context in which to study PA among children [11].

Although the literature on the school built environment and its relationship to PA is well developed [12–20], none of the studies to date have focused on a population of children at high risk of obesity, and few are prospective. Children who are experiencing overweight and obesity may be stigmatized and teased during physical activity and at school [21, 22] making studies that focus on the school environment among a representative sample of school-aged children not necessarily generalizable to youth who are experiencing overweight or obesity. Given that children with overweight and obesity currently represent almost one-third of the Canadian population of 5–17 years [23], this is a critical sub-population that deserves attention so that high-impact leverage points such as schools may be adapted to meet their PA needs. Our aim was therefore to identify potentially salient built, policy/programming and social environmental characteristics in a sample of schools in the Montreal Census Metropolitan Area (MCMA), and then to examine whether these school characteristics are associated with PA, both cross-sectionally and prospectively, among a cohort of youth at high risk of obesity.

## Methods

### Participants and study design

The present study used data from the Quebec Adipose and Lifestyle Investigation in Youth (QUALITY) Cohort Study [24]. Families were recruited using a school-based recruitment strategy. At least one biological parent was required to be with obesity for study inclusion, with the focus being on youth with a parental history of obesity [24]. Among those eligible, 630 families completed baseline data collection between September 2005 and December 2008, including a clinic visit during which questionnaires were completed by both the child and parents and biological and physiological measurements were taken. Questionnaire topics ranged from lifestyle behaviours, the built environment, health and medical history, and socio-demographic details about the kids, parents, and familial relations [24]. A 2-year follow-up assessment was completed in 2008 – 2010. This assessment had many of the same types of data collection, with further details about study design and methodology available in Lambert et al. 2012 which features the full cohort profile [24]. The School Study, an adjunct to the QUALITY Study, contacted 313 schools in the MCMA and conducted detailed audits of 297 schools (95% participation) attended by the study participants residing in the MCMA where principals or physical education teachers were willing to participate. All but one school board participated in this study, and private schools with no school board affiliations were excluded. Analyses are restricted to participants living in the MCMA with complete school-level and reliable accelerometer data at baseline and follow-up. No sex-segregated schools were included in this sample.

#### Application of the conceptual model

The determinants of both children’s PA behaviours and their weight status have been conceptualized using the Social Ecology Model for Health Promotion [25, 26], which has been largely influenced by Bronfenbrenner’s Ecological Systems Theory (EST) [27]. Given that the Social Ecology Model for Health Promotion framework characterises the school environment for PA as one that is multidimensional, we specified three broad domains of the school environment that are likely to have a high impact on student’s PA: 1) the built environment, 2) the policy/programming environment and 3) the social environment. We audited number, variety, and quality of fixed and mobile amenities available for physical activities within the built environment domain. This includes sports and play equipment, including the type and variety of ground covering found in the school yard and gymnasium. We examined school policies described by programs or initiatives for which the aim is to engage students in PA at school. This includes the number and duration of physical education classes, organized and free-play extracurricular activities led by teachers or caregivers during lunch time, before or after school care, including special organized annual PA events such as an “Olympics Day”. Finally, potentially relevant aspects of the school social environment [28] include attitudes, support, and encouragement for PA on the part of peers, the administration, teachers and staff, parents, and the wider community.

## Measures

### Individual-level outcomes and measures

Physical activity was measured using a calibrated accelerometer with an epoch length of 1 min (Actigraph model 7184, Pensacola, Florida, USA) fitted onto the child during the clinic visit and instructed to be worn at the hip for the following 7 consecutive days. Complying with established guidelines [29], data from children with a minimum of 4 days with ≥ 10-h of wear time per day were retained with a minimum of one weekend day measured per participant. Three outcomes were considered in this study using the accelerometer data: (i) mean counts per minute per valid day (MCPM); (ii) mean of daily MVPA (i.e. number of minutes spent in ≥ 2296 CPM/valid day) [30]; (iii) a dichotomized variable for meeting the current Canadian MVPA guidelines [5] of at least 60 min per valid day (i.e. a minimum of 60 min per day spent at ≥ 2296 CPM) versus not. CPM was interpreted according to the established cut-offs of counts per minute [30]: ≤ 100 CPM = sedentary behaviour; > 100 < 2296 CPM = light PA; ≥ 2296 < 4012 CPM = moderate PA, and; ≥ 4012 CPM = vigorous PA.

### Covariates

Child’s age was analyzed as a continuous variable. Pubertal development stage was assessed by a trained nurse using the 5-stage Tanner scales [31, 32]. Puberty was considered either initiated (Tanner stage > 1) or not (Tanner stage = 1) at both baseline and follow-up. Child anthropometrics were measured at baseline and follow-up using standardized protocols [24]. World Health Organization age- and gender- specific body mass index (BMI) z-scores were computed. Children were categorized as overweight or obese if their BMI z-score was greater than 1 standard deviation versus not [33]. Potential seasonal variation in PA was considered as an adjustment variable using the month in which PA was objectively measured. The season variable was dichotomized as accelerometer worn between the months of May and October inclusively, versus not. The highest level of completed education achieved by both parents was reported by the child’s parents at baseline and follow-up. Parental education was categorized as both parents completing secondary education or less, at least one parent with a technical/vocational degree, or at least one parent with a completed university degree. Follow-up analyses used only children who were still attending the same school at follow-up, as well as a secondary analysis including all children at follow-up, regardless of a change in school.

### School-level measures

Measures pertaining to the schools’ built, policy/programming and social environments were gathered by trained research assistants who guided interviews with school principals (82%), physical education teacher (7%), or other teacher (11%) using a questionnaire, as well as conducted direct-observation audits of the schools’ gymnasiums, physical activity equipment and yards at baseline (available upon request). Interviewees responded to questions regarding features of the school policy/programming and social environments that are potentially salient for PA. School interviewees were asked about their school’s PA programs (e.g. the number of PAs offered during the before and after school care programs per week) and aspects of the social environment that may be salient for PA (e.g., the administration’s willingness to promote PA among the students). Other topics included in the questionnaire were general details about the school (e.g. total number of students) and other health initiatives (e.g. extracurricular activities focused on preventing tobacco consumption).

### School built environment

The number, condition, and variety of the school yard’s sports (e.g. soccer goals) and play (e.g. tetherball) related equipment were audited directly by trained research assistants, along with the type and variety of yard ground coverage (e.g. asphalt, grass). Because almost all school yard play equipment was evaluated as in good condition, there was not enough variation to examine these variables regarding the condition of equipment in a meaningful way, and they were not used in subsequent analyses. Six variables, collapsed from the individual school-yard items, were used to evaluate the school yard: 1) total number of sports-related equipment, 2) variety of sports related equipment, 3) low versus high mix of hills, lines and fields in the yard, 4) total number of play-related equipment, 5) variety of play-related equipment, and 6) at least two types of ground covering in the school yard. School gymnasiums were directly audited for the number, variety, and condition of equipment as well as the total diversity of sports-related courts available on the gym floor. Because almost all of the gymnasium equipment was evaluated as in good condition, meaningful analyses of these variables was not possible. Ten variables were derived from the gymnasium audits, combined from the initial data set: 1) variety and 2) sum of fixed gym equipment (e.g. wall ladder, rope), 3) variety and 4) sum of gymnastics related equipment (e.g. balance beam, horse vault), 5) variety and 6) sum of circus related equipment (e.g. unicycle, juggling pins), 7) variety and 8) sum of gym courts (e.g. volleyball basketball), and 9) variety and 10) sum of gym mobile equipment (e.g. basketballs, hula hoops).

### School policy/programming environment

Six variables were created to evaluate potentially salient features of the school’s policy/programming environment, again combined from the initial data set: 1) number of free PAs provided in the school care context/ week, 2) number of free PAs provided in before and after school care/ week, 3) number of free PAs provided outside of the care context/ week, 4) number of special PA events/ year, 5) number of ‘other’ PA events/ per year, and 6) number of school-based PAs/ year.

### School social environment

School principals were asked to answer a 12-item questionnaire about the school social environment. With a response scale for all items retained ranged from 1 = strongly agree to 4 = disagree. Because the distribution was highly positively skewed, responses were dichotomized as strongly agree versus do not strongly agree, which has been employed in other studies [34]. Ten variables with sufficient response variability were included from the questionnaire: 1) administration’s willingness to promote PA among the students, 2) teachers’ willingness to promote PA among the students, 3) caregivers’ willingness to promote PA among the students, 4) parents’ willingness to promote PA among the students 5) community’s is willingness to promote PA among students, 6) student PA leaders present at the school, 7) caregivers given support to enable PA in care contexts, 8) PA offered specifically for girls, 9) parents informed of school activities, 10) dedicated school corridor for safe active transportation to school (i.e. presence of school crossing guards).

### School-neighbourhood variables

Social- and material-level measures of the school neighbourhood environments were obtained at baseline from the Montreal Epidemiological and Geographical Analysis of Population Health Outcomes and Neighbourhood Effects (MEGAPHONE) database [35]. Variables for the proportion of adults age 18–64 years: with no education, employed, living alone, who are separated or widowed, and the average household income, and the proportion of single headed families were calculated for 500-m network buffer zones around the schools. These were combined to create an index of relative material and social deprivation, described by Pampalon et al. [36]. Housing density, calculated as the number of private dwellings per hectare, for the 1000-m network buffer zones around the schools was used as a proxy for urban versus suburban contexts and was dichotomized into the top versus lower two tertiles to represent urban versus suburban environments. The above measures were based on 2006 census data.

## Data analysis

### Principal component analysis

Two principal component (PC) analyses were performed for the built environment domains (yard and gymnasium) and one PC analysis was performed each for the policy/programming and social environments domains on 206 schools with complete data, a total of four analyses. Distinct PCs were conducted to explore the salient features of each area of the school by domain.

### Model specification

We sought to keep models parsimonious by examining unadjusted associations among the covariates and the items describing the school’s environments using generalized estimating equation (GEE) models prior to adjusting the full models. Significant interaction terms between gender and weight status lead us to stratify our analyses by gender. Covariates and PCs were retained if they were associated with the outcome variable of interest at a p ≤ 0.20. Therefore, model specification differs according to each outcome.

We assessed the relation between the PCs and PA by implementing GEE models using PROC GENMOD for both linear and binary outcomes, utilizing an exchangeable correlation structure [37], to account for participants nested within schools. We report 90% confidence intervals, in line with the literature on school features and PA among children [15, 16] and recommendations made in critiques of significance testing [38].

The PCs identified from the yard, gym, policy/programming and social environments of the schools were not correlated; therefore, components were included together in the models. We were interested in whether selected school characteristics are associated with PA over time (rather than a change in PA), therefore we did not adjust for PA measured at baseline. Examination of correlations of MCPM, MVPA and meets the MVPA guidelines found a low to moderate positive correlation (rho = 0.56 for MCPM, rho = 0.58 for MVPA and rho = 0.42 for meets MVPA guidelines) of each measure of PA between baseline and follow-up.

## Results

### Participant characteristics

At baseline, there were 148 girls and 166 boys with complete and reliable data (*n* = 314). At follow-up, there were 54 girls and 75 boys (*n* = 129) still at the same school with reliable accelerometer data. A secondary analysis at follow-up of all participants with school data at baseline and reliable accelerometer data contained 108 girls and 132 boys (*n* = 240). One hundred and twenty-eight participants did not have reliable accelerometer data at follow-up. Table 1 describes individual, familial and PA characteristics of the 314 participants included in the study at baseline and compares them to the 120 with valid accelerometer data from the MCMA excluded due to incomplete school data. Higher rates of attrition occurred among children with overweight and obesity, which may reduce the generalizability of these findings to relevant populations. There were no other significant differences between groups on the variables analyzed at baseline.

**Table 1:** Individual, familial and PA characteristics of the 367 MCMA participants included compared to the 120 MCMA participants excluded* from analyses at Baseline (2005 – 2008)

	**Included in Analyses (*n* = 314)**	**Excluded from analyses (*n* = 120)**	***p*-value**
Age, mean (sd)	9.6 (0.9)	9.6 (0.8)	0.61
Gender (boys), % (n)	52.7 (166)	55.0 (66)	0.69
Puberty initiated (vs uninitiated), % (n)	23.6 (74)	24.2 (29)	0.90
BMI z-score, mean (sd)	0.9 (1.3)	1.2 (1.4)	0.03
Counts per minute, mean (sd)	584.0 (190.5)	586.3 (172.0)	0.91
Minutes of moderate to vigorous PA, mean (sd)	50.3 (26.5)	51.8 (24.3)	0.58
Meets MVPA guidelines (versus not), % (n)	31.2 (98)	34.2 (41)	0.56
PA measured between May and October (vs not), % (n)	54.1 (170)	49.2 (59)	0.35
Both parents with secondary education or less (vs at least one parent with university degree), % (n)	7.3 (23)	9.2 (11)	0.69
At least one parent with technical degree (vs at least one parent with university degree), % (n)	40.8 (128)	37.5 (45)	0.65
At least one parent with university degree (vs not), % (n)	51.9 (163)	53.3 (64)	0.83

*PA* Physical activity

*MVPA* Moderate−to−vigorous physical activity

^*^MCMP Participants excluded because they did not have reliable accelerometer data (≥4 days of minimum 10 h wear)

### Principal components

Two PCs emerged for the school yard environment: 1) *Yard Sports,* and 2) *Yard Unstructured Activities*, that together explain 67% of the variation in the school yard data. Three PCs emerged for the school gymnasiums: 1) *Fixed Gym Equipment*, 2) *Mobile Gym Equipment*, and 3) *Gym Courts*, that together explain 63% of the variation in the gym data. Two PCs emerged for the PA-related policies domain: 1) *Unstructured PAs*, and 2) *Organized PAs*, that together explain 47% of the variation in the PA-related policies. Two PCs emerged for the school social environment: 1) *Willingness to Promote PA*, and 2) *Social Norms to Promote PA*, that together explain 52% of the variation in the social environment data. The minimum eigenvalue retained was 1.10. Internal consistency of components was examined. All variables loaded uniquely and highly onto their respective components at 0.4 or higher, with a minimum loading of 0.43 (Table 2).

**Table 2:** Principal components, variables and variable loadings on principal components

School PA Domain	Principal Component	Variable Description	Variable Loadings^a^ on Principal Components		
**School Yard**			**Yard Sports Features**	**Yard Unstructured Activities**	
	**Yard Sports Features**	Sum of soccer goals, other goals, basketball nets and other nets in yard	**86**	18	
		Variety of sports installations in yard	**86**	20	
		Low or high mix of hills, lines and fields	**66**	11	
	**Yard Unstructured Activities**	Variety of unstructured PA installations in yard	18	**89**	
		Sum of swings, frames, bars, etc. in yard	9	**88**	
		At least 2 types of ground covering in yard (e.g. grass and asphalt)	40	**52**	
**School Gymnasium Features**			**Fixed Gym Equipment**	**Mobile Gym Equipment**	**Gym Courts**
	**Fixed Gym Equipment**	Variety of fixed gym equipment (e.g. wall ladder)	**73**	10	26
		Variety of gymnastics equipment (e.g. vault)	**72**	8	-8
		Sum of fixed gym equipment	**72**	4	29
		Sum of gymnastic equipment	**72**	21	4
	**Mobile Gym Equipment**	Sum of circus equipment (e.g. unicycle)	0	**85**	1
		Variety of circus equipment	5	**69**	-15
		Sum of gym equipment (e.g. bouncy balls)	30	**65**	33
		Variety of gym equipment	33	**60**	34
	**Gym Courts**	Variety of gym courts (e.g. volleyball, basketball)	4	-2	**86**
		Sum of gym courts	22	12	**86**
**School PA -Related Policies and Programs**			**Unstructured PA**	**Organized PA**	
	**Unstructured PA**	Sum of organized unstructured PAs/week	**99**	5	
		Sum of unstructured PA in care context/week	**79**	-9	
		Sum of unstructured PA in before and after school care/week	**71**	-10	
		Sum of unstructured PA/week	**56**	25	
	**Organized PA**	Sum of special PA events/year	-7	**64**	
		Sum of all other types of PA projects/year	13	**61**	
		Sum of school-based PAs	8	**53**	
		Mean minutes of physical education class/week	-8	**43**	
	**High Willingness to Promote PA**	Administration highly willing to promote PA	**79**	27	
		Teachers highly willing to promote PA	**77**	19	
		Caregivers highly willing to promote PA	**75**	26	
		Parents highly willing to promote PA	**71**	12	
		Community highly willing to promote PA	**66**	20	
	**Social Norms in Place to Promote PA**	There are student PA leaders at the school	20	**67**	
		Caregivers are given support for PA	5	**65**	
		There are PAs available specifically for girls	17	**63**	
		Parent are kept informed of PAs	34	**56**	
		There is a school-crossing guard	35	**53**	

^a^Percent of variance in the variable explained by the principal component

## GEE analyses

### Girls’ mean counts per minute

Table 3 presents GEE model estimates for mean accelerometry-based counts per minute among girls at baseline and follow-up. At baseline *Social Norms to Promote PA* and *Unstructured PA Programs* were associated with 32 (90% CI: 13.2, 50.3 MCPM) and 29 (90% CI: 4.4, 53.5 MCPM) more MCPM, respectively in adjusted models. Unadjusted models indicated a positive association between *Social Norms to Promote PA* at follow-up for girls remaining at the same school as at baseline and girls at all schools combined (results not shown), however, when covariates were added to the model this relationship no longer remained significant.

**Table 3:** GEE model estimates for mean accelerometry-based counts per minute among girls at baseline and follow-up (QUALITY cohort school study 2005–2010)

Baseline (2005–2008)					Follow-up Same School as Baseline (2008–2010)		
Parameter	Estimate	90% Confidence Limits				90% Confidence Limits	
				Parameter	Estimate		
Intercept	560.6	333.2	787.9	Intercept	676.6	177.4	1175.7
Age	-4.1	-28.9	20.6	Age	-10.0	-58.9	39.0
Puberty uninitiated ^b^	-107.4^*^	-150.4	-64.5	Puberty uninitiated ^b^	-94.3^***^	-188.5	-0.2
Not overweight or obese ^c^	16.8	-14.0	47.6	Both parents with only high school education^a^	-85.9^*^	-136.5	-35.2
Winter months ^d^	95.6^*^	56.9	134.3	At least 1 parent with college/technical degree^a^	36.2	-33.7	106.1
Social Norms in Place to Promote PA	31.8^**^	13.2	50.3	Material and Social Deprivation Index	-2.4	-6.1	1.2
Unstructured PA Programs	29.0^***^	4.4	53.5	Social Norms in Place to Promote PA	17.6	-17.0	52.2

^a^versus at least 1 parent with a University education

^b^versus puberty initiated

^c^versus overweight or obese

^d^versus summer months

^*^p−value < 0.01

^**^p−value < 0.05

^***^p−value < 0.10

### Girls’ mean daily moderate-to-vigorous physical activity

Table 4 presents GEE model estimates for mean moderate-to-vigorous physical activity among girls at baseline and follow-up. At baseline the *Social Norms to Promote PA* and the *Unstructured PA Programs* PCs were associated with 4.1 (90% CI: 1.8, 6.5 min) and 2.7 (90% CI: 0.4, 4.9 min) more minutes of daily MVPA respectively, while the *Organized PA Programing* PC was associated with 2.1 (90% CI: -3.9, -0.3 min) fewer minutes of daily MVPA. At follow-up among girls who remained at the same school as baseline, the *Fixed Gym Equipment* PC was associated with 5.2 (90% CI: 0.9, 9.5 min) more minutes of daily MVPA. Although, there was a positive relationship between the *Social Norms to Promote PA* and a negative relationship between *Organized PA Programming* and MVPA at follow up, the magnitude of the relationship diminished and no longer remained significant.

**Table 4:** GEE model estimates for mean moderate-to-vigorous physical activity among girls at baseline and follow-up (QUALITY cohort school study 2005–2010)

Baseline (2005–2008)				Follow-up Same School as Baseline (2008–2010)			
Parameter	Estimate	90% Confidence Limits		Parameter	Estimate	90% Confidence Limits	
Intercept	29.5	2.0	57.0	Intercept	36.2	31.8	40.5
Age	0.7	-2.2	3.7	Both parents with only high school education^a^	-18.2^*^	-28.2	-8.1
Puberty uninitiated ^b^	-12.6^*^	-18.0	-7.3	At least 1 parent with college/technical degree^a^	4.9	-3.3	13.1
Winter months _d_	12.5^*^	8.4	16.6	Fixed Gym Equipment	5.2^**^	0.9	9.5
Urban	9.0^*^	4.2	13.7	Organized PA	-3.2	-6.5	0.2
Social Norms in Place to Promote PA	4.1^*^	1.8	6.5	Social Norms in Place to Promote PA	2.1	-1.9	6.2
Unstructured PA Programs	2.7^**^	0.4	4.9				
Organized PA Programs	-2.1^***^	-3.9	-0.3				

^a^versus at least 1 parent with a University education

^b^versus puberty initiated

^d^versus summer months; e) versus suburban context

^*^p−value < 0.01

^**^p−value < 0.05

^***^p−value < 0.10

### Girls’ odds of meeting MVPA guidelines

Table 5 presents the odds of meeting the moderate-to-vigorous physical activity guidelines among girls according to predictors at baseline and follow-up. At baseline, the *Unstructured PA Programs* PC was associated with 2 times the odds (OR: 2.0, 90% CI: 1.3, 7.4) of meeting the daily MVPA guidelines while the *Yard Sports Features* PC was associated with 30% lower odds of meeting the guidelines (OR: 0.70, 90% CI: 0.5, 0.9). Once again, among girls at the same school and all girls combined (results not shown), *Social Norms to Promote PA* was positively, but not significantly associated with odds of meeting the MVPA guidelines at follow-up, although among girls who remained at the same school, this association was borderline significant with 90% confidence.

**Table 5:** Odds of meeting the moderate-to-vigorous physical activity guidelines among girls according to predictors at baseline and follow-up (QUALITY cohort school study 2005–2010)

Baseline (2005–2008)					Follow-up Same School as Baseline (2008–2010)		
Parameter	Estimate	90% Confidence Limits		Parameter	Estimate	90% Confidence Limits	
Not overweight or obese^c^	2.4^***^	1.0	11.4	Age	1.3	0.3	6.3
Puberty uninitiated^b^	0.1^*^	0.0	1.1	Puberty uninitiated^b^	0.1	0.0	1.4
Winter months^d^	3.9^**^	1.4	51.8	Winter months^d^	1.2	0.3	5.3
Urban	6.2^*^	2.4	487.1	Material and Social Deprivation Index	0.9	0.9	1.0
Yard Sports Features	0.7^***^	0.5	1.0	Social Norms in Place to Promote PA	1.8	1.0	3.5
Unstructured PA Programs	2.0^*^	1.3	7.4				

^b^versus puberty initiated

^d^versus summer months

^e^versus suburban context

^*^p−value < 0.01

^**^p−value < 0.05

^***^p−value < 0.10

### Boys’ mean counts per minute

Table 6 presents GEE model estimates for mean counts per minute among boys at baseline and follow-up. At baseline, the *High Willingness to Promote PA* PC was associated with 40 (90% CI: 10.3, 70.4 MCPM) more MCPM, while the *Yard Sports* PC was associated with 23 (90% CI: -40.4, -5.9 MCPM) fewer MCPM among boys. At follow-up among boys who remained at the same school as at baseline, the *Fixed Gym Equipment* and *High Willingness to Promote PA* PC was associated with 21.5 (90% CI: 0.7, 42.2 MCPM) and 44.8 (90% CI: 5.1, 84.4) more MCPM, respectively. Among boys at all schools at follow-up (*n* = 132), the *Yard Sports* PC was associated with 38 fewer (90% CI: -56.1, -19.9) MCPM (results only shown here).

**Table 6:** GEE model estimates for mean counts per minute among boys at baseline and follow-up (QUALITY cohort school study 2005–2010)

Baseline (2005–2008)					Follow-up Same School as Baseline (2008–2010)		
		90% Confidence Limits				90% Confidence Limits	
Intercept	946.7	688.8	1204.6	Intercept	501.6	453.4	549.9
Age	-46.7^*^	-72.9	-20.5	Puberty uninitiated^b^	-103.3^*^	-160.7	-45.9
Not overweight or obese^c^	139.6^*^	92.2	186.9	Not overweight or obese^c^	149.8^*^	95.8	203.8
Winter months^d^	84.6^*^	40.5	128.6	Fixed Gym Equipment	21.5^***^	0.7	42.2
Yard Sports Features	-23.1^**^	-40.4	-5.9	High Willingness to Promote PA	44.8^***^	5.1	84.4
High Willingness to Promote PA	40.4^**^	10.3	70.4				

^b^versus puberty initiated

^c^versus overweight or obese

^d^versus summer months

^*^p−value < 0.01

^**^p−value < 0.05

^***^p−value < 0.10

### Boys’ mean daily moderate-to-vigorous physical activity

Table 7 presents GEE model estimates for mean moderate-to-vigorous physical activity among boys at baseline and follow-up. At baseline, the *Yard Unstructured Activities Features* and *High Willingness to Promote PA* PCs were associated with 3.6 (90% CI: 0.2, 6.9 min) and 5.6 (90% CI: 1.2, 10 min) more minutes of MVPA, respectively. At follow-up among boys at the same school as baseline and among boys at all schools, the *High Willingness to Promote PA* was associated with 7.3 (90% CI: 1.5, 13.2) and 4.5 (90% CI: 0.8, 8.3) more minutes of MVPA, respectively (results only shown here for boys at all schools).

**Table 7:** GEE model estimates for mean moderate-to-vigorous physical activity among boys at baseline and follow-up (QUALITY cohort school study 2005–2010)

Baseline (2005–2008)				Follow-up Same School as Baseline (2008–2010)			
Parameter	Estimate	90% Confidence Limits		Parameter	Estimate	90% Confidence Limits	
Intercept	83.4	47.7	119.2	Intercept	44.1	37.5	50.7
Age	-4.4^**^	-8.0	-0.8	Puberty uninitiated^b^	-12.9^*^	-20.9	-4.9
Not overweight or obese^c^	21.1^*^	14.2	28.1	Not overweight or obese^c^	22.8^*^	14.8	30.8
Winter months^d^	10.5^**^	4.0	16.9	High Willingness to Promote PA	7.3^**^	1.5	13.2
Yard Unstructured Activities Features	3.6^***^	0.2	6.9				
High Willingness to Promote PA	5.6^**^	1.2	10.0				

^b^versus puberty initiated

^c^versus overweight or obese

^d^versus summer months

^*^p−value < 0.01

^**^p−value < 0.05

^***^p−value < 0.10

### Boys’ odds of meeting MVPA guidelines

Table 8 presents odds of meeting the moderate-to-vigorous physical activity guidelines according to predictors among boys at baseline and follow-up. At baseline, the *High Willingness to Promote PA* PC was associated with 2 times the odds of meeting the MVPA guidelines (OR: 2.0, 90% CI: 1.5, 2.8) whereas the *Yard Sports Features* PC was associated with 30% fewer odds (OR: 0.70, 90% CI: 0.6, 0.9) of meeting the guidelines. At follow-up, among boys remaining at the same school as at baseline and among all boys, the *High Willingness to Promote PA* was associated with 130% higher odds (OR: 2.3, 90% CI: 1.1 – 4.7) and 80% higher odds (OR: 1.8, 90% CI: 1.1, 2.9) of meeting the MVPA guidelines, respectively (results only shown here for boys at all schools).

**Table 8:** Odds of meeting the moderate-to-vigorous physical activity guidelines according to predictors among boys at baseline and follow-up (QUALITY cohort school study 2005–2010)

Baseline (2005–2008)					Follow-up Same School as Baseline (2008–2010)		
Parameter	Estimate	90% Confidence Limits		Parameter	Estimate	90% Confidence Limits	
Age	0.7^***^	0.5	0.9	Not overweight or obese^c^	6.2^*^	2.3	16.5
Not overweight or obese ^c^	5.4^*^	2.8	10.5	Winter months^d^	2.4	1.0	5.6
Winter months ^d^	2.0^***^	1.1	3.5	High Willingness to Promote PA	2.3^***^	1.1	4.7
Yard Sports Features	0.7^**^	0.6	0.9				
High Willingness to Promote PA	2.0^*^	1.5	2.8				

^b^versus puberty initiated

^c^versus overweight or obese

^d^versus summer months

^*^p−value < 0.01

^**^p−value < 0.05

^***^p−value < 0.10

## Discussion

This study identified nine conceptually unique multidimensional features of schools that are potentially salient for PA based on their built, policy/programming and social environments among a population at high risk of obesity. Seven of the nine school domains identified were associated with PA at baseline and at follow-up, particularly for boys. Girls who attended school environments that were high on the *Social Norms in Place to Promote PA* PC were more physically active. The variable “PAs available specifically for girls” loaded onto the *Social Norms in Place to Promote PA* PC. This may mean that girls who attended schools that provided activities that responded to their interests were more physically active. Boys who attended schools where there was a very high willingness to promote PA were more physically active. Both girls and boys who attended schools where there were opportunities for unstructured PAs, and yard sports installations were more physically active.

This is the first study to our knowledge to 1) identify conceptually unique multidimensional features of schools based on multiple domains using PC analysis and to 2) assess existing school features (as opposed to an intervention) and PA among a group of children at risk of obesity. Although previous research has evaluated physical [12–15, 17–20], policy [15, 16, 20] and/or social [15, 16] features of the school environment, only two others [15, 16] have assessed features of built environment, policy/programming, and social characteristics of schools and their associations with PA. Neither of these studies were longitudinal and they did not stratify by gender.

The *Yard Sports Features* PC was inversely associated with MCPM for boys at both baseline and follow-up, and it was negatively associated with odds of meeting MVPA guidelines among boys and girls at baseline. A recent systematic review of the school environment and adolescent PA found an indeterminate relationship between PA facilities or areas/fields and PA [39] among a younger sample. Our results suggest that yard sports features may not be supportive of meeting physical activity guidelines among children at high risk of obesity. Our null findings at follow-up regarding meeting the MVPA guidelines suggests that any negative effect of *Yard Sports Features* on children’s physical activity does not last. Together with the positive association observed between *Yard Unstructured PA Features* and boys’ MVPA at baseline, these results suggest that features of schoolyards may be more important for boys’ PA than for girls.

Gym context and equipment are rarely evaluated in studies of school features and PA. The few studies that examined access to sports or PA equipment in schools [19, 40, 41] found they were not associated with PA or were associated with PA only among boys. Our results agree in part. We found fixed and gymnastics equipment (that make up the *Fixed Gym Equipment* PC) were associated with PA outcomes at follow-up among girls and boys. It may be that fixed gym installations and gymnastics equipment are attractive for PA among children who are exposed for least two years.

The *Organized PA Programs* PC was associated with fewer minutes of MVPA among girls at baseline. Although counterintuitive, these results are not unprecedented. A previous study found that children at high risk of obesity are more likely to be physically active in non-sports promoting environments [42], and boys have been found to partake in more free, unstructured play activities than girls [43]. Additionally, other studies have largely demonstrated a lack of association between minutes of Physical Education and PA [44–47]. We also observed that *Unstructured PA Programs* were important for all the girls’ PA outcomes at baseline, indicating that it is unstructured rather than organized PA programs that are likely important for girls, at younger ages. Contextual opportunities for free unstructured play in schools, such as the presence of green areas and play structures have been positively associated with objectively measured PA [14–16, 20], and our results point to the importance of allocating time for unstructured activities for girls’ PA levels as well.

The school social environment may be particularly important for girls’ and boys’ PA, although in different ways. We have not come across any studies on school features and PA that have asked schools whether there are student PA leaders or PAs available specifically for girls, two variables that load onto the *Social Norms in Place to Promote PA* PC. Given that girls are less physically active than boys [48], it may be important for schools to incorporate student PA leaders and provide activities specifically for girls. Studies have observed that girls’ MVPA, but not boys’, is associated with distance to school [43] and walkable and woodland environments [18, 49]. Thus, the presence of walkable school-transit corridors (e.g. presence of a school-crossing guard) may be another component in girls’ cumulative daily MVPA and MCPM.

*High Willingness to Support PA* was consistently associated with the three PA outcomes for boys at both baseline and follow-up. Literature suggests that among those who are highly willing to promote PA, it may be the teachers’ willingness that is the most important. A review of studies of the school environment and PA [39] found that perceived teacher’s support for PA was consistently positively associated with PA, as opposed to adult supervision, which was consistently not associated with PA, both genders combined.

Together, these results speak to concerns in the literature about the limitations of emphasizing sport, adult-led, and structured activities for meeting children’s PA needs [50–52]. Although organized PAs may be easier to implement, encourage and study, they have been shown to explain very little in the variation of children’s MVPA [52]. In addition, it has been suggested that an emphasis on encouraging children to engage in supervised or adult-led, structured physical activities rather than encouraging free, unsupervised, or unstructured play to meet the MVPA guidelines may be contributing, in part, to the decline in fitness among Canadian children [53] and a decrease in PA among children more generally [54, 55]. In addition, these results suggest some promising implications for cost-effective interventions for schools wishing to increase PA levels among their students. Schools are, as expected, high-interest leverage points for children’s health and health behaviours [7]. There is still a significant lack of literature about children at risk of obesity in a school setting, meaning there are plenty of opportunities to understand this particular population and design appropriate interventions to promote MVPA and promote healthy habits. With such significant rates of obesity in the population, including children and teenagers [23], most if not all children can be considered at risk of obesity.

Although only cross-sectional results were significant for girls, they suggest that schools have the potential to increase girls’, specifically girls at risk of obesity’s, mean MVPA by 9 min per day if there are social norms in place to promote PA along with unstructured PA programs and an absence of organized PA programs. Nine additional minutes of MVPA represents 15% of the total daily MVPA minutes girls require daily to meet the MVPA guidelines. Schools with social norms in place to promote PA and unstructured PA programs may increase girls’ MCPM by 62. Sedentary behaviour is being ≤ 100 CPM, thus increasing MCPM by 62 is substantial. Increasing PA promotion and having yard unstructured activity features in school may increase the mean MVPA for boys at risk of obesity by 10 min per day, representing 17% of the daily MVPA guidelines. Boys’ MCPM may increase by 63 in schools where there are no yard sports installations and a high willingness to promote PA.

### Study strengths and limitations

Study strengths include the use of a socio-ecological framework to guide the conceptualisation of study design and development of the PCs, with multiple and conceptually distinct domains within schools. Physical activity was objectively measured which increases confidence in the reliability of study outcomes. School built environment features were also measured by an external, trained observer, reducing the potential for reporting bias. Finally, we assessed the relationship between school features and PA prospectively, adding strength to the study design and our evaluation of the relationships observed.

There were a number of study limitations. The study contained few reliable accelerometer data and a high number of students who changed or aged out of baseline schools which limited our follow-up results. Owing to the small sample size, the study results may have lacked power for some analyses, particularly for modeling girls PA outcomes at follow-up. We used binary data in our PC analyses which downgrades the solution, however, this method can still provide a worthwhile description of the relationships in a set of variables [56]. Our more generous alpha level may limit comparability with studies that used a more restrictive alpha, however the use of an alpha level of 0.10 is not uncommon in studies of built environment features and children’s PA outcomes [15, 16, 42].

The Principal or Physical Education teacher responses may be subject to social desirability bias, and thus may have limited our ability to assess some of their responses on a more refined scale (rather than “very highly willing” versus not). PA measures were not limited to PA during school hours; however, we were interested in how school features affected overall PA. School features, particularly social environmental features at schools may have an effect on PA both within and outside of the school environment given that schools can be considered high leverage points for students’ behaviour. Although we measured a large number of school features, we may have failed to identify other, unmeasured but salient features; further investigation to confirm and expand these findings is necessary. None of the schools included in this study were sex-segregated, and private schools, due to their lack of affiliation to a school board, were not included in this study. Both are missing populations from this research. Finally, this is an exploratory study with data-driven analyses, which is useful for helping to guide further research.

The study provides new insights into multidimensional and conceptually distinct features of schools for PA among children at high risk of obesity that can be pursued in future studies. These should consider associations between school features and sedentary behaviour in addition to light PA and MVPA, as it remains unclear if school features associated with a decrease in MCPM, or MVPA are subsequently associated with an increase in sedentary behaviour.

This study may be generalizable to children who are experiencing overweight, obesity, or are at high risk of being so due to a parental history of obesity. Nevertheless, given that almost one-third of the Canadian youth population are experiencing overweight or obesity, this is an important sub-population of youth who represent a sizable proportion of the population that may have a disproportionate disease burden both during childhood and into adulthood. Future studies will be necessary to confirm the direction and magnitude of effects and to establish their relevance to health promotion efforts.

## Conclusions

Our findings demonstrate the multidimensional nature of school features that may be salient for PA, and the complex relationships between school social and built environments and PA among girls and boys at high risk of obesity. Findings provide ample evidence to support further research in larger samples for physical activity amenities, programming and physical activity promotion efforts within schools.

## Data Availability

Data are available from the corresponding author on reasonable request.

## Abbreviations

PA: Physical activity
MVPA: Moderate-to-vigorous physical activity
PC: Principal Components
MCMA: Montreal Census Metropolitan Area
QUALITY: Quebec Adipose and Lifestyle Investigation in Youth
EST: Ecological Systems Theory
MCPM: Mean counts per minute
CPM: Counts per minute
BMI: Body mass index
MEGAPHONE: Montreal Epidemiological and Geographical Analysis of Population Health Outcomes and Neighbourhood Effects
GEE: Generalized estimating equation
